# Relationships between starch synthase I and branching enzyme isozymes determined using double mutant rice lines

**DOI:** 10.1186/1471-2229-14-80

**Published:** 2014-03-26

**Authors:** Natsuko Abe, Hiroki Asai, Hikari Yago, Naoko F Oitome, Rumiko Itoh, Naoko Crofts, Yasunori Nakamura, Naoko Fujita

**Affiliations:** 1 Department of Biological Production, Akita Prefectural University, Akita City, Akita 010-0195, Japan

**Keywords:** Amylopectin, Amylose, Branching enzyme, Mutant, Rice, Starch synthase

## Abstract

**Background:**

Starch is the most important carbohydrate in plant storage tissues. Multiple isozymes in at least four enzyme classes are involved in starch biosynthesis. Some of these isozymes are thought to interact and form complexes for efficient starch biosynthesis. Of these enzyme classes, starch synthases (SSs) and branching enzymes (BEs) play particularly central roles.

**Results:**

We generated double mutant lines (*ss1/be1* and *ss1*^*L*^*/be2b*) between SSI (the largest component of total soluble SS activity) and BEI or BEIIb (major BEs in developing rice endosperm) to explore the relationships among these isozymes. The seed weight of *ss1/be1* was comparable to that of wild type, although most *ss1/be2b* seeds were sterile and no double recessive plants were obtained. The seed weight of the double recessive mutant line *ss1*^*L*^*/be2b*, derived from the leaky *ss1* mutant (*ss1*^*L*^) and *be2b*, was higher than that of the single *be2b* mutant. Analyses of the chain-length distribution of amylopectin in *ss1/be1* endosperm revealed additive effects of SSI and BEI on amylopectin structure. Chain-length analysis indicated that the BEIIb deficiency significantly reduced the ratio of short chains in amylopectin of *ss1*^*L*^*/be2b*. The amylose content of endosperm starch of *ss1/be1* and *ss1*^*L*^*/be2b* was almost the same as that of wild type, whereas the endosperm starch of *be2b* contained more amylose than did that of wild type. SSI, BEI, and BEIIb deficiency also affected the extent of binding of other isozymes to starch granules.

**Conclusions:**

Analysis of the chain-length distribution in amylopectin of the double mutant lines showed that SSI and BEI or BEIIb primarily function independently, and branching by BEIIb is followed by SSI chain elongation. The increased amylose content in *be2b* was because of reduced amylopectin biosynthesis; however, the lower SSI activity in this background may have enhanced amylopectin biosynthesis as a result of a correction of imbalance between the branching and elongation found in the single mutant. The fact that a deficiency of SSI, BEI, or BEIIb affected the affinity of other starch biosynthetic isozymes for the starch granule implies that there is a close interaction among SSI, BEI and BEIIb during amylopectin biosynthesis in rice endosperm.

## Background

Starch, one of the most important life-sustaining carbohydrates, consists of two homopolymers of the α-D-glucosyl unit; amylopectin and amylose. Amylopectin is a large branched molecule and has a molecular weight greater than 10^8–10^ Da, while amylose is a linear molecule. The amylopectin and amylose content strongly affect the physical and chemical properties of starch. At least four classes of enzymes are involved in starch biosynthesis: ADP-glucose pyrophosphorylase (AGPase), starch synthase (SS), starch branching enzyme (BE), and starch debranching enzyme (DBE) [1-4]. Among these four enzyme classes, SSs and BEs have central roles in starch biosynthesis.

SS (EC 2.4.1.21) elongates α-glucans by adding a Glc residue from ADP-Glc to the non-reducing ends of α-glucans via α-1,4 glucosidic linkages. Among all of the starch biosynthesis enzymes, SS has the most isoforms [5,6]. *SSI*, *SSIIa*, *SSIIIa*, and *GBSSI* genes are strongly expressed in developing rice endosperm [5,6]. The first three SS isozymes are involved only in amylopectin biosynthesis, whereas GBSSI is involved in biosynthesis of amylose and extra-long chains of amylopectin [7].

SSI is the largest component of total soluble SS activity in developing rice endosperm. SSI-deficient mutants (*ss1*) were isolated from an SSIIa-inactive Japonica rice cultivar Nipponbare by reverse genetics via insertion of the retrotransposon *Tos17*[8]. The changes in amylopectin chain-length distribution were examined in four *ss1* allelic mutants, in which *Tos17* was inserted into the *SSI* gene in different positions. These mutants exhibited different levels of SSI activity (0%–25% that of wild type) that were positively correlated with the degree of changes in amylopectin chain-length distribution. Chain-length distribution analyses indicated that SSI generates the degree of polymerization (DP) 8–12 chains from very short (DP 6–7) chains emerging from the branch point of the A and B_1_ chains of amylopectin [8]. This was confirmed by an *in vitro* experiment using recombinant rice SSI [9]. Surprisingly, the *ss1* mutation did not affect the size and shape of seeds or starch granules, or the crystallinity of endosperm starch. Nevertheless, SSI accounts for more than 60% of soluble SS activity in developing rice endosperm. SSIIIa, which is the second largest component of total soluble SS activity in developing rice endosperm, elongates long chains that connect amylopectin clusters [10]. A double recessive homozygous mutant derived from *ss1* and *ss3a* null mutants was sterile; however, double recessive mutant lines containing a leaky *ss1* mutant and a *ss3a* null mutant were fertile, as were mutant lines in which one or both of these genes were heterozygous with the wild-type allele. These results suggested that SSI and/or SSIIIa are required for starch biosynthesis in rice endosperm [11].

BE (EC 2.4.1.18) is the only enzyme that can introduce α-1,6 glucosidic linkages into α-glucans. Therefore, it plays an essential role in amylopectin biosynthesis. Higher plants have two types of BE; BEI and BEII. Rice and maize have two BEII isoforms, BEIIa and BEIIb. BEIIb has only been detected in the endosperm, whereas BEI and BEIIa have been detected in all organs. The seed phenotype and starch accumulation of a BEI-deficient mutant were equivalent to those of wild type, but the chain-length distribution of amylopectin differed. In the BEI-deficient mutant, there were reduced levels of long chains with DP ≥ 37 and short chains with 12 ≤ DP ≤ 21, but increased levels of short chains with DP ≤ 10 and 24 ≤ DP ≤ 34 [12]. In contrast to the relatively mild phenotypic differences between the BEI-deficient mutant and wild type, the characteristics of endosperm starch differed substantially between BEIIb-deficient mutant lines and wild type. BEIIb-deficient mutants accumulated significantly fewer amylopectin short chains with DP ≤ 13, resulting in strong resistance to gelatinization [13].

Biochemical analyses of BEI-deficient [12] and BEIIb-deficient [13] mutant lines and *in vitro* analyses of recombinant BEI and BEIIb [14,15] suggested that the two enzymes have different roles. BEI is involved in transferring longer chains that eventually link multiple clusters of amylopectin with the medium size chains in the amylopectin amorphous lamellae. In contrast, BEIIb specifically transfers the short chains that represent the border between the amorphous lamellae and the crystalline lamellae of amylopectin [3,15].

Recent analyses of protein–protein interactions in maize have strongly suggested that SSI, SSIIa, and BEIIb interact during starch biosynthesis [16,17]. SS and BE isozymes in developing rice endosperm likely co-operate with each other during starch biosynthesis. Analyses of double mutant lines with mutations in genes encoding SS and BE isozymes is one strategy to further analyze the interactions among these enzymes and their functions.

In this study, we generated double recessive mutants from deficient (*ss1*) or leaky (^L^) *ss1* mutants (*ss1*^*L*^) and BEI- or BEIIb-deficient mutants (*ss1/be1* and *ss1*^*L*^*/be2b*). The structure and components of starch differed between the double mutant lines and the parental mutant lines. The pleiotropic effects of the deficient isozyme activities and their relationships in starch biosynthesis in rice developing endosperm are discussed.

## Results and discussion

### Generation of double recessive mutant lines between SSI and BEs

**Figure 1:**
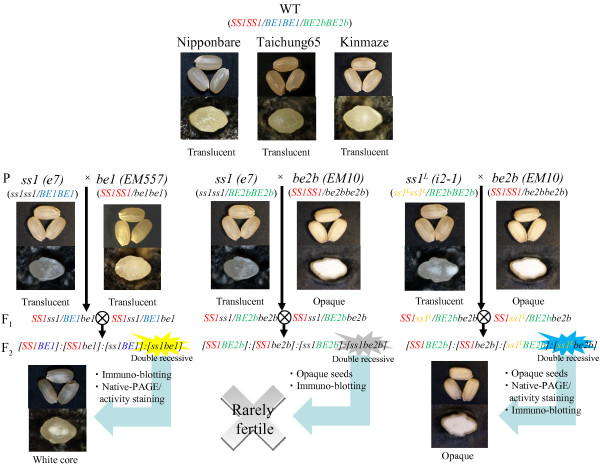
**Pedigree and seed morphologies of double mutant lines.** Morphology of dehulled rice seeds (upper panels) and seed cross-sections (lower panel). Seeds were obtained from double mutant lines derived from SSI and BEI or BEIIb mutants and their parent lines, and were observed under a stereo-microscope. Symbols in parentheses show genotypes of lines.

To generate a double mutant line (*#4011*) between SSI and BEI, the *ss1*-null mutant (*e7*) [8] was crossed with the *be1*-null mutant (*EM557*) [12]. The resulting double recessive F_2_ seeds were identified by immunoblotting, and showed a translucent or white-core phenotype (Figure 1). The F_3_ seeds generated by self-pollination of double recessive F_2_ plants were segregated into translucent (~28%) and white-core (~72%) phenotypes, although this ratio varied among different growth years. The seeds of both phenotypes of F_2_ and F_3_ seeds were deficient in SSI and BEI, and the chain-length distribution patterns of amylopectin from both phenotypes were identical (data not shown). Therefore, segregation of the seed phenotype was independent of the *SSI* and *BEI* genotype. The difference in seed phenotypes may be related to environmental conditions (data not shown). Therefore, two phenotypes, translucent and white-core seeds of *ss1/be1*, were combined and used as the *ss1/be1* double mutant for further analyses.

To generate a double mutant line between SSI and BEIIb, the *ss1*-null mutant (*e7*) [8] was crossed with the *be2b*-null mutant (*EM10*) [13]. One quarter of the F_2_ seeds were opaque, indicative of BEIIb deficiency, and were screened by immunoblotting. Approximately 2% (2 out of the 88 opaque F_2_ seeds screened) were double recessive for both SSI and BEIIb, indicating that most of the double recessive seeds were sterile. Unfortunately, none of the seedlings from double recessive opaque seeds survived (Figure 1). The fact that the double recessive mutant seeds between SSI and BEIIb were rarely fertile indicated that in rice, SSI and BEIIb are indispensable isozymes for both endosperm starch biosynthesis and plant development.

To produce a non-sterile double recessive mutant line between SSI and BEIIb, we used the leaky *ss1* mutant line *i2-1*[8] as the *ss1* mutant parental line in a cross with the *be2b*-null mutant line (*EM10*). Double recessive F_2_ seeds (*#4017*) were selected from opaque seeds by immunoblotting. The growth and yield of *#4017* were less than half those of wild type (data not shown). The seed weight of *#4017* was approximately 70% that of wild type (Table 1). These results suggested that even the small amount of SSI activity in *#4017* was sufficient to avoid sterility in the absence of BEIIb.

**Table 1:** Dehulled grain weight, starch content, and apparent amylose in rice mutant lines

**Line**	**Genotype**	**Background**	**Grain weight**^**a **^**(mg)**	**Starch content**^**b **^**(mg)**	**Amylose content**^**c **^**(mg/seed)**
Nipponbare (Nip)	Wild type (WT)	Nip	20.7 ± 0.2 (100.0)^d^	14.7 ± 0.1 (100.0)^d^	3.13
Taichung 65 (T65)	WT	T65	20.6 ± 0.2 (100.0)^e^	13.3 ± 1.0 (100.0)^e^	2.94
*e7*	*ss1*	Nip	19.0 ± 0.2^*,**^ (91.7)^d^	13.7 ± 3.5 (93.1)^d^	3.19
*EM557*	*be1*	T65	23.4 ± 0.1^*,**^ (112.7)^e^	14.5 ± 0.7 (108.9)^e^	3.15
*#4011*	*ss1/be1*	Nip/T65	21.0 ± 0.2 (101.4)^d^	12.5 ± 1.5 (85.1)^d^	2.68
Kinmaze (Kin)	WT	Kin	20.5 ± 0.2 (100.0)^f^	13.6 ± 0.4 (100.0)^f^	2.92
*i2-1*	*ss1*^*L*^	Nip	21.2 ± 0.2^**^ (102.4)^d^	11.9 ± 1.5 (80.8)^d^	2.50
*EM10*	*be2b*	Kin	11.7 ± 0.2^*,**^ (56.4)^f^	6.2 ± 0.5^*^ (45.1)^f^	1.82
*#4017*	*ss1*^*L*^*/be2b*	Nip/Kin	14.2 ± 0.2^***^ (68.5)^d^	8.7 ± 2.0^***^ (59.3)^d^	1.88

^a^Mean ± SE of 50 seeds.

^b^Mean ± SE of three seeds.

^c^Amylose content per seed = Starch content^b^ × apparent amylose content (%) (Table 3).

^d^Percentage of wild type (Nipponbare).

^e^Percentage of wild type (Taichung 65).

^f^Percentage of wild type (Kinmaze).

^*^Significant difference between single mutant and wild-type lines (*t*-test, P < 0.05).

^**^Significant difference between parental mutant and double mutant lines (*t*-test, P < 0.05).

^***^Significant difference between double mutant and wild-type lines (*t*-test, P < 0.05).

F_3_ and F_4_ seeds from *#4011* (*ss1/be1*) and *#4017* (*ss1*^*L*^*/be2b*) double recessive mutant lines were used for further analyses.

### Seed weight and starch content

Of the three BE isozymes expressed in rice endosperm, BEIIb is the major isozyme required for branching of amylopectin molecules. Indeed, a deficiency in BEIIb induced a more pronounced phenotype than did a deficiency in either BEI or BEIIa [3,12,13]. A deficiency of SSI or BEI activity did not affect seed phenotype or starch accumulation [8,12]. The dehulled grain weight and starch content of *ss1/be1* seeds were not significantly different from those of wild type and parent mutant lines (Table 1). By contrast, the dehulled grain weight and starch content were greatly reduced in *be2b* seeds, to 56% and 45% of the levels in wild type, respectively (Table 1) [13]. The dehulled grain weight and starch content were higher in the *ss1*^*L*^*/be2b* double mutant than in the single *be2b* mutant, and were 69% and 59% of the levels in wild type, respectively. It is unclear why starch accumulation was higher in the double mutant than in the single mutant.

In *be2b*, there were significantly fewer non-reduced ends of amylopectin molecules. This led to a decrease in starch accumulation because of an imbalance between branching by BEs and elongation by SSs. Excess chain elongation may lead to steric inhibition within the amylopectin cluster structure. In the double mutant lines between BEIIb and SSI (*ss1*^*L*^*/be2b*), this excessive chain elongation and imbalance may have been countered by reduced SSI activity, leading to greater starch accumulation than that in the single *be2b* mutant.

### Pleiotropic effects of SSI and BEs deficiencies on other starch biosynthesis enzymes

**Figure 2:**
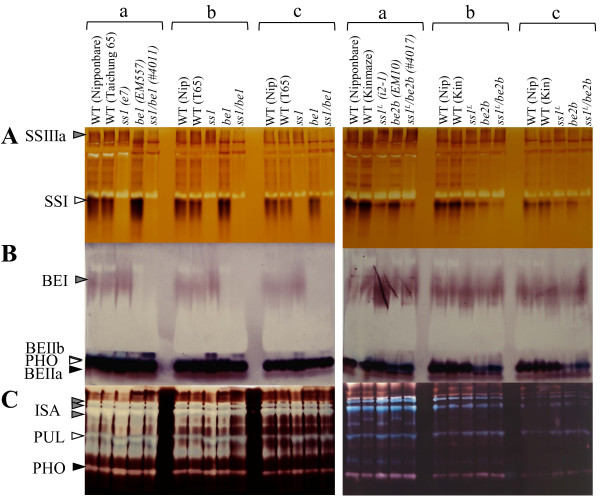
**Enzyme activities on zymograms.** Native-PAGE/activity staining of starch synthases (SSs; Panel **A**), branching enzymes (BEs; Panel **B**), and debranching enzymes (DBEs; Panel **C**) in developing endosperm of double mutant, parental mutant, and wild-type lines. Arrowheads indicate SSI, SSIIIa, BEI, BEIIa, BEIIb, ISA (isoamylase), PUL (pullulanase), and PHO (phosphorylase) activity bands. Soluble protein extracts were prepared from developing endosperm. Amount of soluble protein is per mg fresh weight. Volumes of crude extract applied to the native gels in section ‘a’ are 2-fold and 4-fold greater than those applied in sections ‘b’ and ‘c’, respectively. Kin, Kinmaze; Nip, Nipponbare; T65, Taichung 65; WT, wild type.

**Figure 3:**
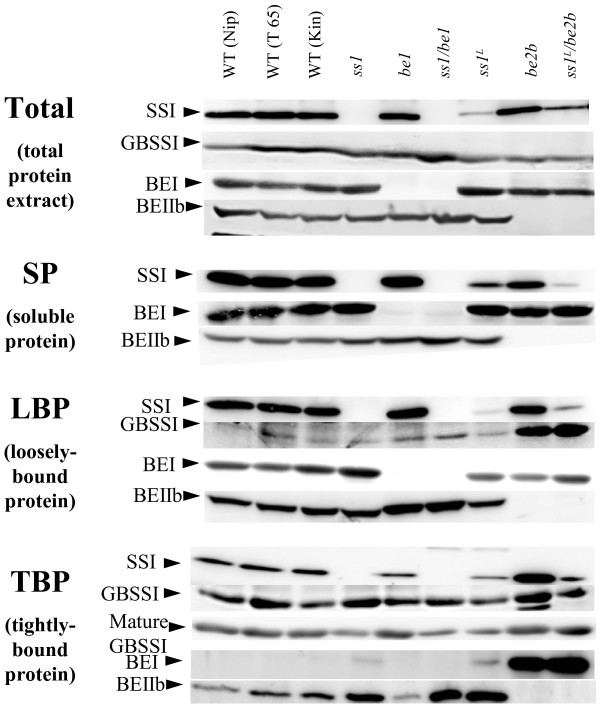
**Distributions of enzymes in total protein extract and three protein fractions.** Immunoblotting of total protein extract (Total), soluble protein fraction (SP), loosely-bound protein fraction (LBP), and tightly-bound protein fraction (TBP) from three developing rice endosperms at 12–15 days after flowering (DAF). Samples are from double mutant, parental mutant, and wild-type lines. Immunoblotting analyses were conducted using antisera raised against rice SSI, GBSSI, BEI, and BEIIb. GBSSI from mature endosperms (mature GBSSI) were also analyzed. Amounts of protein loaded onto the gels were as follows: 1/25 of extracts in SP, 1/15 of extracts in LBP and 1/4 of extracts in TBP. Amount of protein bands in ‘Total’ was standardized against density of total protein bands stained with Coomassie Brilliant Blue (data not shown). Kin, Kinmaze; Nip, Nipponbare; T65, Taichung 65; WT, wild type.

To confirm that *ss1, be1, and be2b* single mutant and double mutant lines were deficient in the relevant proteins and enzyme activities, we conducted zymogram analyses by semi-quantitative native- polyacrylamide gel electrophoresis (PAGE) (Figure 2) and immunoblotting assays (Figure 3; Total and SP) of the soluble protein fraction (SP) from developing endosperm at 12 days after flowering (DAF). SSI activity was completely absent from *ss1* and *ss1/be1* mutants, and the SSI activity in *ss1*^*L*^ mutants was less than one-third that in wild type (Figures 2A and 3). SSI activity and the amount of SSI protein in the SP were approximately 50% lower in *be2b* than in wild type. Decreases in SSI activity and total SS activity in *be2b* (*EM10*) were similarly demonstrated in previous studies on rice [13] and maize [18]. These results support the hypothesis that SSI mainly elongates short chains (DP 6–7) that are branched by BEIIb [15], and that a close interaction among the isozymes is required during starch biosynthesis [16]. SSI activity and the amount of SSI protein in the SP were approximately 30% lower in *ss1*^*L*^*/be2b* than in *ss1*^*L*^ (Figures 2A and 3; SP). The intensity of the SSI activity band in *ss1*^*L*^*/be2b* was estimated at 5%–10% of that in wild type according to the semi-quantitative zymogram (Figure 2A). This small amount of SSI activity is one possible explanation for the fertility of *ss1*^*L*^*/be2b*, although other possibilities cannot be excluded. The BEI activities in SSI-deficient or SSI-reduced lines were slightly higher than that in wild type (Figure 2B). The activities of other starch biosynthesis isozymes, namely SSIIIa (Figure 2A), BEIIa (Figure 2B), ISA, PUL, and PHO1 (Figure 2C), were not significantly different between the mutant lines and wild type.

To clarify whether the increased starch content in the *ss1*^*L*^*/be2b* double mutant was because of enhanced substrate levels, AGPase activity in developing seeds at 10-15 DAF were quantified (Table 2). The synthesis of ADP-glucose by AGPase is the first committed step, and the rate-limiting step, for starch biosynthesis. Previous studies showed that mutant lines with high amylose contents often show elevated AGPase activities [10,11]. The results are shown in two different units, per microliter of extract and per endosperm, so the data is comparable to previous studies. The results of AGPase activity per microliter extract showed that the AGPase activity was significantly higher in the *be2b* single mutant than in its parental line. The AGPase activity of *ss1*^*L*^*/be2b* was also significantly higher than in *ss1*^*L*^. This suggests that loss of BEIIb enhances the AGPase activity. There were no significant difference in AGPase activity between the double mutant lines (*ss1/be1* and *ss1*^*L*^*/be2b*) and wild types (Table 2). The value of AGPase activity per endosperm was also calculated by multiplying the total volume of extract obtained from the endosperm. This result reflects the weight of endosperm. *ss1/be1* showed highest total AGPase activity as the endosperms used for this experiment were heavier.

**Table 2:** AGPase activity in developing seeds (DAF 10–15) of double mutants, their parental lines, and wild type

**Lines**	**nmol min**^**-1**^ **μl**^**-1**^^**a**^	**μmol min**^**-1 **^**endosperm**^**-1a**^
Wild type	17.7 ± 0.4 (100)^b^	0.550 ± 0.016 (100)^b^
(Nipponbare)		
Wild type	16.9 ± 0.2 (100)^c^	0.550 ± 0.016 (100)^c^
(Taichung 65)		
*ss1*	18.2 ± 0.4 (103)^b^	0.628 ± 0.017 (114)^b, *^
(*e7*)		
*be1*	16.8 ± 0.3 (99)^c^	0.533 ± 0.019 (97)^c, **^
(*EM557*)		
*ss1/be1*	17.9 ± 0.2 (101)^b^	0.726 ± 0.071 (132)^b, ***^
(#4011)		
Wild type	17.0 ± 0.3 (100)^d^	0.566 ± 0.027 (100)^d^
(Kinmaze)		
*ss1*^*L*^	16.7 ± 0.8 (94)^b, **^	0.551 ± 0.022 (100)^b, **^
(*i2-1*)		
*be2b*	19.3 ± 0.5 (113)^d, *^	0.689 ± 0.026 (122)^d, *^
(*EM10*)		
*ss1*^*L*^*/be2b*	19.4 ± 0.9 (109)^b^	0.660 ± 0.040 (120)^b^
(*#4017*)		

^a^Mean ± SE. n = 4 for all the lines except for #4017 (n = 2).

^b^Percentage of the wild type Nipponbare.

^c^Percentage of the wild type Taichung 65.

^d^Percentage of the wild type kinmaze.

*Significant differences between single mutant and wild-type lines (*t*-test, P < 0.05).

**Significant differences between parental mutant and double mutant lines (*t*-test, P < 0.05).

***Significant differences between double mutant and wild-type lines (*t*-test, P < 0.05).

To measure the expression levels of proteins encoded by genes for the main isozymes (*SSI*, *GBSSI*, *BEI* and *BEIIb*) in starch biosynthesis, the amounts of these proteins were compared by immunoblotting using total proteins extracted with urea buffer (Figure 3; Total). The amount of GBSSI protein was higher in *ss1/be1* than in other lines. The amount of BEI protein was also slightly higher in *ss1* and *ss1*^*L*^ than in wild type. These results indicated that the deficiency in some starch biosynthetic enzymes led to elevated levels of other starch biosynthetic enzymes.

Next, we determined the localization of these extra starch biosynthetic enzymes. We prepared a SP fraction representing amyloplast stroma, a loosely-bound protein (LBP) fraction, and a tightly-bound protein (TBP) fraction in which proteins are bound to starch granules. Then, the amount of proteins related to starch biosynthesis in each fraction were compared by immunoblotting.

We examined the pleiotropic effects of deficiencies in SSI, BEI, and BEIIb on various protein fractions from developing endosperm. SSI, GBSSI, BEI, and BEIIb were detected by immunoblotting (Figure 3; LBP and TBP). The amount of SSI in the SP fraction was higher in *ss1*^*L*^ than in *ss1*^*L*^*/be2b*, and vice versa in the LBP and TBP fractions. In wild type, BEI was detected in the SP and LBP fractions, but not in the TBP fraction. Although there were low levels of BEI in the TBP fraction in *ss1* and *ss1*^*L*^, there were much higher levels of BEI in the TBP fraction in *be2b* and *ss1*^*L*^*/be2b.* There was slightly more BEI in the LBP fraction from *ss1* than in the same fraction from other lines (Figure 3; LBP). In the LBP fraction, there was slightly more BEIIb in BEI-deficient mutant lines (*be1* and *ss1/be1*) than in the other lines (Figure 3; LBP).

In the TBP fraction, the amount of BEIIb was higher in SSI-deficient (*ss1* and *ss1/be1*) and SSI-reduced lines (*ss1*^*L*^) than in the other lines (Figure 3; TBP). Dense SSI bands were detected in the TBP of *be2b*. Previous studies reported similar results from maize *ae* (BEIIb-deficient) mutant lines [16]. These results suggested that deficiencies in BEIIb in rice and maize affect the binding of SSI and BEI to starch granules. Both stromal and granule-bound BEI and BEIIb in maize are phosphorylated, and these phosphorylation events play an important role in the formation of the protein–protein complex during amylopectin synthesis [17]. The absence of BEIIb in maize resulted in the recruitment of other starch biosynthetic enzymes to the starch biosynthetic protein complex and to starch granules. Therefore, a deficiency of SSI, BEI and BEIIb rice isozymes may have altered the binding of other starch biosynthetic isozymes to the starch granules in rice, as is the case in maize. It is possible that the deficiency of a specific isozyme is compensated for by other isozymes to allow the formation of starch biosynthetic protein complexes.

SSI was distributed in every fraction (SP, LBP, and TBP) in maize [19] and rice [8]. SSI in developing rice endosperm tends to bind to starch granules at later stages [8], indicating that this binding occurs as dehydration progresses. One explanation for the increased amount of SSI in the TBP fraction in BEIIb-deficient lines is that the abundance of long-chain amylopectin molecules in the *be2b* background lines could lead to the early maturation and dehydration of endosperm (Asai *et al.*, in preparation). It could be that more SSI binds to starch granules in developing endosperm (12 DAF) in *be2b* background lines than in other lines.

Binding of GBSSI to starch granules was also affected by BEIIb deficiency. We detected dense bands corresponding to GBSSI in the LBP fraction of *be2b* and *ss1*^*L*^*/be2b*, but only faint bands in wild type, *ss1*, *be1*, *ss1/be1,* and *ss1*^*L*^. By contrast, the amount of GBSSI (per mg starch) in the TBP of developing and mature endosperm was comparable between *ss1*^*L*^*/be2b* and wild type (Figure 3; TBP). This might be related to the similar amylose contents in *ss1*^*L*^*/be2b* and wild type (Table 3).

### Characterization of starch structure in mature endosperm of double mutant lines

**Figure 4:**
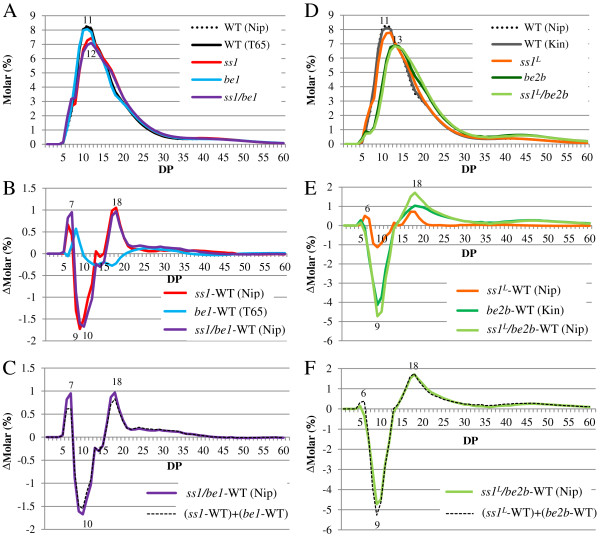
**Molecular structure analysis of amylopectin by capillary electrophoresis.** Chain-length distribution patterns of endosperm amylopectin in mature endosperm **(A, D)**. Differential plots between single mutant and wild-type lines **(B, E)**. Differential plots between double mutant and wild type and the calculated profiles made by adding the profiles of single mutant lines **(C, F)**. Numbers on plots represent DP values. Each figure shows one representative data set (one of at least three replicates prepared from different rice seeds from homogenous plants). Relative standard error of molar% of each chain length from DP5–60 was less than 2.5%. Kin, Kinmaze; Nip, Nipponbare; T65, Taichung 65; WT, wild type.

Next, we compared the structure of amylopectin among the double mutant lines, their parental mutant lines, and wild-types. We analyzed the endosperm amylopectin isoamylolysate chain-length distribution by capillary electrophoresis (Figure 4). The results showed that the chain-length distribution patterns of *ss1/be1* were similar, but not identical, to those of *ss1* (Figure 4A and B). The profile obtained for *ss1/be1* was almost identical to that of the calculated profile made by adding the profiles of the *ss1* and *be1* single mutants (Figure 4C). However, in *ss1/be1*, the amount of chains with DP 7 and DP 18 was higher, and the amount of chains with DP 9 was lower, compared with those in the calculated profile. This indicated that there was an additive effect of the reduction of SSI activity on the chain-length of amylopectin in the *be1* background, with a slight synergistic enhancement.

The chain-length distribution patterns of *ss1*^*L*^*/be2b* were similar, but not identical, to those of the *be2b* mutant line (Figure 4D and E). Compared with *be2b, ss1*^*L*^*/be2b* had significantly fewer short chains with DP ≤ 13 and more long chains with DP ≥ 14. The chain-length profile of *ss1*^*L*^*/be2b* was almost identical to that calculated by adding the profiles of the *ss1*^*L*^ and *be2b* single mutants (Figure 4F), except that the molar (%) of very short chains with DP 6–7 did not increase in the pattern generated from *ss1*^*L*^*/be2b-*WT profile. The molar% of DP 6–7 chains was lower in *ss1*^*L*^*/be2b* (Figure 4D) than in wild type as a result of BEIIb deficiency. These results indicated that there was an additive effect of the reduction of SSI activity on the chain-length of synthesized amylopectin in the *be2b* background. This finding strongly suggests that the reaction proceeds from branch formation by BEIIb to chain elongation by SSI. If there are no or only a few branches generated by BEIIb in the crystalline lamellae, then SSI could lose its main function, since branched chains are the preferred target chains for elongation by SSI.

**Figure 5:**
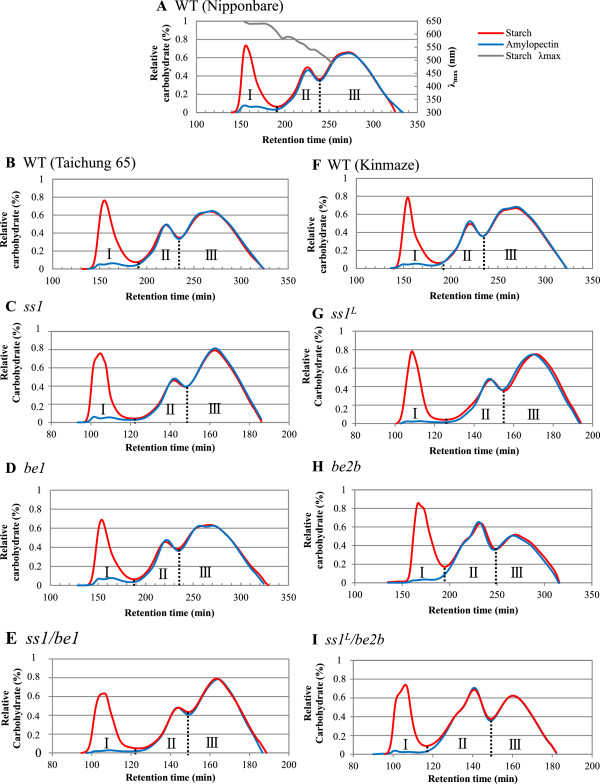
**Size separation of debranched endosperm starch and purified amylopectin by gel-filtration.** Size separation of debranched endosperm starch and purified amylopectin from double mutant **(E and I)**, parental mutant **(C, D, G and H)**, and wild-type lines **(A, B and F)** by gel filtration chromatography through three Toyopearl HW55S-HW50S columns. Graphs show elution profiles of isoamylase-debranched starch (blue lines) and purified amylopectin (red lines). Different X-axis scales reflect different flow rates among experiments. Fractions (Fr. I, II, and III) were divided at troughs of carbohydrate content curve, as detected by refractive index detectors (left Y-axis). Grey line in wild type (Nipponbare) indicates λ_max_ values of starch-iodine complexes (right Y-axis). Figures show one typical dataset (one of at least three replicates prepared from starch and purified amylopectin).

To further investigate the components of starch and its structure in the double mutant lines, the isoamylolysates of endosperm starch and purified amylopectin were subjected to size-exclusion chromatography using Toyopearl HW55S and HW50S columns (Figure 5). The λ_max_ values greater than 600 nm, which represent the α-glucan-iodine complex, indicated that fraction I (Fr. I) contained most, if not all, of the amylose (Figure 5A). A small amount of carbohydrate was also detected in Fr. I from a purified amylopectin sample (Figure 5). This was “extra-long chain” (ELC, DP ≥ 500) amylopectin [7,20]. Therefore, Fr. I from endosperm starch contained both true amylose and ELC. The value obtained by subtracting the ELC content from the apparent amylose content (AAC) of starch is equivalent to the true amylose content (TAC) of starch [20]. Fr. II included B_2_-_3_ long chains of amylopectin connecting 2–3 clusters of amylopectin, while Fr. III included short chains within a single cluster of amylopectin. The proportion of each starch component was calculated based on the data shown in Figure 5. The results are shown in Table 3.

**Table 3:** Composition of carbohydrate (weight%) in endosperm starch fractions separated by gel filtration chromatography

**Lines**		**Fr. I (%)**^**a**^	**Fr. II (%)**	**Fr. III (%)**	**TAC (%)**^**e**^	**III/II**
Wild type	Starch^b^	21.3 ± 0.3^d^	20.4 ± 0.0	58.5 ± 0.3	18.2	2.9 ± 0.0
(Nipponbare)	Amylopectin^c^	3.0 ± 0.4	17.7 ± 0.9	54.4 ± 1.8	-	3.1 ± 0.1
Wild type	Starch	22.1 ± 0.8	20.6 ± 0.0	57.4 ± 0.9	19.0	2.8 ± 0.0
(Taichung 65)	Amylopectin	2.8 ± 0.1	19.4 ± 0.0	57.7 ± 0.1	-	3.0 ± 0.0
*ss1*	Starch	23.3 ± 0.4^*,**^	18.7 ± 0.4^*^	58.0 ± 0.1	20.8^*^	3.1 ± 0.1
(*e7*)	Amylopectin	2.5 ± 0.2^**^	18.6 ± 0.5	58.2 ± 4.5	-	3.1 ± 0.2
*be1*	Starch	21.7 ± 1.2	19.5 ± 0.4	58.8 ± 1.5	18.1	3.0 ± 0.1
(*EM557*)	Amylopectin	3.6 ± 0.1^*,**^	19.0 ± 0.3	58.4 ± 1.2	-	3.1 ± 0.1
*ss1/be1*	Starch	21.4 ± 0.3	20.5 ± 0.8	58.1 ± 0.6	20.0	2.9 ± 0.1
(#4011)	Amylopectin	1.4 ± 0.1^***^	20.2 ± 0.9	57.6 ± 1.1	-	2.9 ± 0.2
Wild type	Starch	21.5 ± 1.9	21.2 ± 0.7	57.3 ± 1.9	19.2	2.7 ± 0.2
(Kinmaze)	Amylopectin	2.3 ± 0.1	21.4 ± 0.5	59.6 ± 2.2	-	2.8 ± 0.2
*ss1*^*L*^	Starch	21.0 ± 0.3	21.8 ± 0.5^*,**^	57.2 ± 0.3^*,**^	19.7	2.6 ± 0.1^*,**^
(*i2-1*)	Amylopectin	1.3 ± 0.0^*^	21.2 ± 0.5^*,**^	59.1 ± 2.6^**^	-	2.8 ± 0.0^*,**^
*be2b*	Starch	29.4 ± 0.8^**^	34.3 ± 0.6^*^	36.3 ± 0.4^*^	28.1^*,**^	1.1 ± 0.0^*^
(*EM10*)	Amylopectin	1.3 ± 0.1^*^	35.8 ± 1.4^*^	38.9 ± 1.5^*^	-	1.1 ± 0.0^*,**^
*ss1*^*L*^*/be2b*	Starch	21.6 ± 0.6	37.5 ± 1.2^***^	40.9 ± 1.9^***^	19.7	1.1 ± 0.0^***^
(*#4017*)	Amylopectin	1.2 ± 0.1^***^	37.8 ± 0.5^***^	38.6 ± 1.0^***^	-	1.0 ± 0.0^***^

^a^Three fractions (Fr. I, II and III) were divided at troughs of carbohydrate content curve, as detected by refractive index detectors (Figure 5).

^b^Total carbohydrate content = 100%.

^c^Areas of Fr. II and Fr. III of amylopectin were superimposed onto those of the starch, and amount of amylopectin (extra-long chain) in Fr. I was calculated.

^d^Mean ± SE of three replicates.

^e^True amylose content (TAC) = apparent amylose content (Fr. I of starch) – extra-long chains (Fr. I of amylopectin).

*Significant differences between single mutant and wild-type lines (*t*-test, P < 0.05).

**Significant differences between parental mutant and double mutant lines (*t*-test, P < 0.05).

***Significant differences between double mutant and wild-type lines (*t*-test, P < 0.05).

The proportions of AAC in *ss1* (23.3%), *be1* (21.7%) and *ss1/be1* (21.4%) were slightly higher than, or similar to, those in the Nipponbare wild type (21.3%) and Taichung 65 wild type (22.1%). This result indicated that deficiencies in SSI and/or BEI did not greatly affect amylose content (Figure 5 and Table 3), although the TAC of *ss1* was significantly higher than that of wild type.

There was a greater proportion of ELC in *be1* (3.6%) than in wild type, but a smaller proportion of ELC in *ss1/be1* (1.4%) than in wild type. The ratio of Fr. III to Fr. II (III/II) in endosperm starch was similar in *ss1/be1* (2.9) and the parental mutant and wild types (2.8–3.1) (Table 3).

The proportion of AAC was similar in *ss1*^*L*^ (21.0%) and wild-type Nipponbare (21.3%), whereas that in *be2b* (29.4%) was significantly higher than that in wild-type Kinmaze (21.5%). Interestingly, the proportion of AAC in *ss1*^*L*^*/be2b* (21.6%) was similar to that in wild type. This suggested that the trend of BEIIb deficiency to increase amylose content was counteracted by a reduction in SSI activity.

The proportion of AAC was approximately 1.5-fold higher in the SSIIIa-deficient mutant (*ss3a*, ca. 30%) than in wild type (ca. 20%) [10]. Amylose content per seed was higher in *ss3a* (2.8 mg) than in wild type (2.1 mg) [11]. The GBSSI content, especially starch granule-bound GBSSI (GBSSI in TBP), is related to amylose content [11,21]. The presence of a single nucleotide polymorphism in exon four of *OsGBSSI* reduced binding of GBSSI to starch granules and resulted in a low amylose content [22]. The results from those reports implied that functional GBSSI is distributed in the TBP, where it can synthesize amylose. A deficiency in SSIIIa enhances *GBSSI* expression [10], resulting in an increase in GBSSI and thus, more AAC than in wild type. In the present study, the proportion of AAC was 1.4-fold higher in *be2b* (29.4%) than in wild type (21.5%). The amount of GBSSI in TBP (per mg starch) was higher in *be2b* than in wild-type Kinmaze (Figure 3; TBP)*.* On the other hand, amylose content per seed was much lower in *be2b* (1.82 mg) than in wild type (2.92 mg) because of the reduced starch accumulation in *be2b* (Table 1). These data suggested that the mechanism underlying the increase in AAC in *be2b* differs from that of *ss3a*. While the amylose content per seed was similar in *ss1*^*L*^*/be2b* and *be2b* (Table 1), the proportion of AAC was much lower in *ss1*^*L*^*/be2b* than in *be2b* (Table 3 and Figure 5). Thus, there was greater starch accumulation in *ss1*^*L*^*/be2b* than in *be2b* (Table 1) because of the increase in amylopectin biosynthesis caused by the reduction in SSI activity in the *be2b* background.

The ratio of Fr. III to Fr. II (III/II) in endosperm starch was similar in *ss1*^*L*^ (2.6) and wild type (2.9) (Table 3). By contrast, the Fr. III to Fr. II ratios in *be2b* and *ss1*^*L*^*/be2b* (1.1) were significantly lower than that in wild type. These results are consistent with the chain-length distribution patterns determined by capillary electrophoresis (Figure 4D and E), indicating that BEIIb deficiency was responsible for the significant reduction in the amount of short chains with DP ≤ 13.

## Conclusions

We generated double recessive mutant lines of SSI and BE isozymes (BEI and BEIIb) and analyzed their starch phenotypes. The complete deficiency of both SSI and BEIIb led to a significant reduction in fertility, and the seeds could not grow into plantlets. However, starch accumulation was recovered in double mutants in which BEIIb was absent but SSI was reduced (*ss1*^*L*^*/be2b*). These results suggest that in rice, SSI and BEIIb are indispensable isozymes for both starch biosynthesis in the endosperm and plant development, and only a small amount of SSI activity is sufficient for fertility. By contrast, starch accumulation and AAC were not affected in lines deficient in both SSI and BEI. Structural analyses of starch in the double mutant lines reveal additive effects of SSI and BEI on amylopectin structure. The effects of SSI and BEIIb on amylopectin structure were also primarily additive, except that the levels of very short chains with DP 6-7 were reduced in *ss1*^*L*^*/be2b* and *be2b* and increased in *ss1*^*L*^, compared with wild type. These results strongly suggest that branch formation by BEIIb is followed by chain elongation by SSI. The increased AAC in *be2b* was because of a reduction in amylopectin biosynthesis, whereas an additional reduction of SSI activity against a background of BEIIb deficiency might enhance amylopectin biosynthesis via resolving the imbalance between branching by BEs and chain elongation by SSs.

The results also showed that deficiencies in SSI, BEI and BEIIb affected the starch granule-binding of the other isozymes. Although the precise mechanisms remain unclear, these data strongly support a close interaction among SSI, BEI, and BEIIb during amylopectin biosynthesis in rice endosperm.

Liu *et al.*[17] showed that in maize, at least SSI, SSIIa, and BEIIb form a protein complex in the stroma with a phosphorylation dependent manner [17] and that these protein–protein complexes are trapped in starch granules during starch biosynthesis. They also described that granule-bound BEI and BEIIb in maize endosperm were completely phosphorylated [17]. They hypothesized that these complexes are the functional components involved in amylopectin clusters [16]. Therefore, a deficiency of rice SSI, BEI and BEIIb isozymes may have altered the binding of other starch biosynthetic isozymes to the starch granules in rice, as is the case in maize. According to our preliminary immuno-precipitation experiments using developing endosperm of Japonica rice, it is likely that at least SSI, BEI, and BEIIb form complexes in the SP fraction. Although we have no evidence that these complexes are functional in the LBP or TBP fractions, it is possible that other isozymes replace deficient ones, as shown in this study. For example, the significant increase in amylopectin long chains with DP ≥ 14 in *be2b* and *ss1*^*L*^*/be2b* (Figure 4D, E) might result not only from the deficiency of BEIIb, but also from its replacement by BEI, which functions in the branching of long chains [15], in the TBP fraction (Figure 3).

One of the most widely accepted amylopectin structure models to date is the “cluster model” proposed by Hizukuri [23]. This model proposes that clusters interconnect through long amylopectin chains that participate in forming the crystalline and amorphous lamellae. Bertoft [24] proposed an alternative “two-directional backbone model”. This model proposes that long chains of amylopectin are not integrated as parts of the clusters, and the clustered chains are orientated perpendicular to the direction of the amorphous backbone. However, currently it is unknown which model represents true amylopectin structure.

Understanding the interactions and relationships among starch biosynthetic enzymes during starch biosynthesis will help to clarify the architecture of amylopectin structure. Further analyses of isozyme complexes in mutant lines, including double mutant rice lines, are required to answer these and other questions.

## Methods

### Plant materials

The mutant *ss1, be1,* and *be2b* lines were used in crosses. We used two *ss1* lines; *e7,* an SSI-deficient mutant (*ss1*), and *i2-1,* an SSI-leaky mutant (*ss1*^*L*^) [8], as well as the *be1* and *be2b* mutant lines (BEI- and BEIIb-deficient mutants *EM557*[12] and *EM10*[13], respectively). As wild-type controls, we used the cultivars Nipponbare, which is the parent of *e7* and *i2-1*, Kinmaze, which is the parent of *EM10*, and Taichung 65, which is the parent of *EM557*. Three combinations of double mutant lines (*ss1* × *be1*, *ss1* × *be2b*, and *ss1*^*L*^ × *be2b*) were isolated and the resulting double heterozygotes (F_1_) were self-pollinated (Figure 1). In the cross between *ss1* and *be1*, double recessive F_2_ seeds (*ss1/be1*) were selected after immunoblotting analyses of the mature endosperm. In the crosses between *ss1* or *ss1*^*L*^ and *be2b*, double recessive F_2_ seeds (*ss1/be2b* and *ss1*^*L*^*/be2b*) were selected by the opaque seed phenotype and by immunoblotting of the mature endosperm. Rice plants were grown during summer in an experimental paddy field at Akita Prefectural University, Japan, under natural environmental conditions.

### Protein extraction from developing and mature endosperm

Total proteins were extracted from developing endosperm from three individuals (12 DAF) per line using 200 μL urea buffer [125 mM Tris–HCl (pH 6.8), 8 M Urea, 4% (w/v) SDS and 5% (v/v) β-mercaptoethanol] and a plastic pestle. The homogenate was incubated with a rotator (Iuchi MTR-103, Japan) at 37°C for 2 h. The homogenate was centrifuged at 20,000 *g* at room temperature for 20 min and the supernatant was set aside. The pellet was homogenized in 200 μL urea buffer, and then centrifuged under the same conditions. The pooled supernatants were loaded on SDS-PAGE and the proteins were stained with Coomassie Brilliant Blue (CBB).

The amounts of total proteins were normalized to the same intensity of general protein bands on SDS-PAGE gel stained with CBB, and used for immunoblotting. SP and LBP fractions were prepared from developing and mature endosperm as follows: seeds were ground in extraction buffer [50 mM imidazole-HCl (pH 7.4), 8 mM MgCl_2_, 50 mM 2-mercaptoethanol, and 12.5% (v/v) glycerol] to obtain the SP fraction. The resulting pellet after centrifugation at 20,000 *g* for 10 min at 4°C was extracted with SDS solution [55 mM Tris–HCl (pH 6.8), 10% SDS, 5% 2-mercaptoethanol, and 12.5% (v/v) glycerol] to obtain the LBP fraction. The TBP fraction was extracted from the pellet after boiling with SDS solution for 7 min as described in a previous report [8].

### Native-PAGE/activity staining and immunoblotting

Native-PAGE/activity staining of DBE and BE was performed as previously described [25,26]. The activity bands corresponding to ISA [27], PUL [28], PHO1 [29], BEI [12], BEIIb [13], and BEIIa (unpublished data) were previously identified using mutant lines .

Native-PAGE/SS activity staining was performed on 7.5% (w/v) acrylamide slab gels containing 0.8% (w/v) oyster glycogen (G8751, Sigma), according to Nishi *et al.*[13], with the addition of 0.5 M citrate.

We conducted immunoblotting analyses for four extracts (Total, SP, LBP and TBP) as previously described [30]. We used antiserum raised against SSI (5,000 times dilution for total protein extract, 1,000 times dilution for SP, 1,500 times dilution for LBP, and 1,000 times dilution for TBP) [8]; antiserum raised against BEI (4,000 times dilution for total protein extract, 1,000 times dilution for SP, 1,000 times dilution for LBP, and 1,000 times dilution for TBP) [12]; antiserum raised against BEIIb (5,000 times dilution for total protein extract, 2,000 times dilution for SP, 2,000 times dilution for LBP, and 750 times dilution for TBP) [13], and antiserum raised against GBSSI (4,000 times dilution for total protein extract, 500 times dilution for LBP, and 1,000 times dilution for TBP) [10].

### AGPase activity assay

AGPase activity was quantified as described in a previous report [31]. Soluble protein was extracted from developing rice endosperms at DAF 10–15 using three volumes grinding solution. Extract was diluted one hundred fold. Then, 30 μL supernatant was mixed with 220 μL assay mixture (100 mM Hepes-NaOH, pH 7.4, 3 mM ADP-glucose, 3 mM Sodium pyrophosphate, 3.7 mM 3-phosphoglycerate, 5 mM MgCl_2_, 5 mM DTT). The reaction was carried out at 30°C for 20 min and terminated by boiling. The samples were diluted with 350 μL water and then centrifuged. Then, 10 μL 10 mM NADP^+^ was added to a 500 μL aliquot of the supernatant. After addition of 0.2 U phosphoglucomutase and 1 U glucose 6-phosphate dehydrogenase, the absorbance at 340 nm was measured using a spectrophotometer. The amount of NADPH was calculated using the extinction coefficient 6.22 mmol^-1^ cm^2^.

### Analysis of starch and amylopectin structure

Starch was extracted from mature rice endosperms to assess the amylopectin chain-length distribution as described by Fujita *et al.*[32]. The chain-length distributions of endosperm α-glucans were analyzed using capillary electrophoresis methods [32,33] using a P/ACE MDQ Carbohydrate System (Beckman Coulters, CA, USA).

Gel filtration chromatography of starches and amylopectin was performed as previously described [10,28] using a Toyopearl HW55S gel filtration column (300 × 20 mm) connected in series to three Toyopearl HW50S columns (300 × 20 mm) equipped with an RI (refractive index) detector (Tosoh RI-8020).

## Abbreviations

AAC: Apparent amylose content; ADP: Adenosine diphosphate; BE: Branching enzyme; DAF: Days after flowering; DBE: Debranching enzyme; DP: Degree of polymerization; DTT: Dithiothreitol; ELC: Extra-long chain; Fr: Fraction; GBSSI: Granule-bound starch synthase I; Glc: Glucose; LBP: Loosely-bound protein; PAGE: Polyacrylamide gel electrophoresis; SDS: Sodium dodecyl sulfate; SP: Soluble protein; SS: Starch synthase; TBP: Tightly-bound protein.

## Competing interests

The authors declare that they have no competing interests.

## Authors’ contributions

NA performed seed weight measurements, native-PAGE/activity staining, immunoblotting, gel filtration, and chain-length distribution analyses. HA and HY assisted with gel filtration and chain-length distribution analyses. NFO performed starch content measurements. RI assisted with screening of double recessive mutant lines. NC measured AGPase activity and helped to summarize data and write the manuscript. YN helped to write the manuscript. NF performed crosses between single mutant lines, wrote the manuscript, and planned, supervised, and coordinated the project. All authors read and approved the final manuscript.
